# Electroacupuncture Alleviates Pain Responses and Inflammation in Collagen-Induced Arthritis Rats via Suppressing the TLR2/4-MyD88-NF-*κ*B Signaling Pathway

**DOI:** 10.1155/2023/9050763

**Published:** 2023-02-04

**Authors:** Shi-Yue Sun, Qi-Qi Yan, Li-Na Qiao, Yi-Nan Shi, Lian-Hong Tan, Yong-Sheng Yang

**Affiliations:** Institute of Acupuncture and Moxibustion, China Academy of Chinese Medical Sciences, Beijing 100700, China

## Abstract

**Results:**

EA intervention and OxPAPC injection could relieve mechanical allodynia and thermal hyperalgesia caused by CIA. Paw edema and pathological damage of synovium were significantly ameliorated after EA intervention and OxPAPC injection. Furthermore, EA intervention and OxPAPC injection markedly reduced the contents of serum TNF-*α*, IL-1*β*, and IL-6, as well as the protein expression levels of synovial TLR2, TLR4, MyD88, and NF-*κ*B p-p65. In particular, the expression of TLR2 and TLR4 on synovial fibroblasts and macrophages in synovium was significantly reduced by EA intervention.

**Conclusions:**

Repeated EA stimulation at ST36 and SP6 can effectively relieve joint pain and synovial inflammation caused by RA in CIA rats. The analgesic and anti-inflammatory effect of EA may be closely related to the inhibition of innate immune responses driven by the TLR2/4-MyD88-NF-*κ*B signaling pathway in the synovium.

## 1. Introduction

Rheumatoid arthritis (RA) is a chronic autoimmune polyarticular disease that can affect all ages, genders, and ethnicities [1]. RA has a worldwide prevalence of 0.5%–1% and is clinically characterized by marked synovial inflammation, joint swelling, joint pain, bone erosion, and progressive disability [2]. It severely degrades the sufferers' quality of life and is even life-threatening [3]. Existing antirheumatic drugs, including nonsteroidal anti-inflammatory drugs (NSAIDs), disease-modifying antirheumatic drugs (DMARDs), or bio-agents, only partially halt the RA progression and have many adverse effects [4, 5]. Growing evidence suggests that acupuncture, one of the nonpharmacological alternative therapies, is effective on RA [6–8]. It was reported that acupuncture significantly improved disease activity scores, pain and overall mobility, joint swelling, and health-related quality of life in patients with RA [9–11]. Although it is generally accepted that EA can be used as adjuvant therapy for the treatment of RA, its precise mechanisms remain to be elucidated.

RA is the consequence of dysregulation of the immune system and is characterized by an excessive autoimmune response to synovial membrane. The process involves the activation of synoviocytes, inflammatory cells infiltration in synovium, and the increase of pro-inflammatory cytokines, such as tumor necrosis factor-alpha (TNF-*α*), interleukin 1 beta (IL-1*β*), and interleukin 6 (IL-6), leading to chronic localized synovial and systemic inflammation, joints pain, as well as cartilage and bone erosion [12]. At present, it is widely accepted that the cytokine network plays a vital role in RA pathogenesis [13], and anticytokines therapy in patients with RA has proven to be effective [14]. Moreover, EA has been previously reported to reduce the serum levels of pro-inflammatory cytokines in RA patients [15] and RA animal models [16, 17], which suggests the possible immunomodulatory mechanism of EA in alleviating RA.

Toll-like receptors (TLRs) are functionally key pattern recognition receptors (PRRs) in the innate immune system, which are mainly expressed in the innate immune cells and play a critical role in the initiation of inflammatory responses [18]. Numerous studies have demonstrated that TLRs and their downstream signaling molecules are involved in the development of autoimmune diseases such as RA [19, 20]. The human TLRs family includes 11 members, mainly distributing in cell membranous structures. Among them, TLR2 and TLR4 are closely associated with the pathogenesis of RA [18, 21]. Additionally, growing in vivo and in vitro studies have emphasized that TLR2 and TLR4 signaling play pivotal roles in the onset and maintenance of synovial inflammatory responses in RA [22–25]. However, it remains unknown whether the TLR2/4 signaling is the regulatory target through which EA exerts its antiarthritic effects on RA.

In the present study, we established a collagen-induced arthritis (CIA) model in rats to investigate the ameliorative effects of EA on RA synovitis and arthralgia through TLR2/4 and their downstream signaling cascades. Our findings highlighted the importance of the synovial TLR2/4-MyD88-NF-*κ*B signaling pathway in the analgesic and anti-inflammatory effects of EA intervention for RA.

## 2. Materials and Methods

### 2.1. Animals

Animal care and experimental protocols were approved by the Ethics Committee of the Institute of Acupuncture and Moxibustion, China Academy of Chinese Medical Sciences (ethical approval number: D2017-08-16-1). All experimental animals were cared according to the guidelines provided by the U.S. National Institutes of Health for the Care and Use of Laboratory Animals. 200–250 g male Sprague–Dawley (SD) rats were purchased from Beijing Union Medical College and were housed (up to 5/cage) in standard plastic cages with water and standard mouse feed under controlled temperature (22–24°C), humidity (55 ± 5%), and 12-hr alternating light/dark cycle (lights were turned on between 7:00 a.m. and 7:00 p.m.).

### 2.2. Establishment of the CIA Model

The CIA model was established according to reference [26–28]. Briefly, bovine type II collagen (CII; Chondrex, Redmond, WA, USA) (dissolved in 0.1 M acetic acid) was emulsified 1 : 1 (v: v) with complete Freund's adjuvant (CFA; Sigma-Aldrich, Darmstadt, Germany) at a final concentration of 1 mg/mL. Rats were immunized with intradermal injection of the 300 *μ*l CII emulsion at the base of the tail on day 0, followed by the same booster injection 7 days later. The animals with no signs of swelling in joints were removed. Controls were handled in the same way except that they were injected with 150 *μ*l of 0.1 M acetic acid.

### 2.3. Experiment Design

#### 2.3.1. Experiment 1

To determine the analgesic and anti-inflammatory effects of EA treatment on the CIA rats, we randomly divided 36 SD rats into three groups: control (normal), CIA (model only), and CIA + EA (model with EA treatment at ST36 and SP6 acupoints) (*n* = 12 each group).

#### 2.3.2. Experiment 2

To assess the role of the TLR2/TLR4 signaling pathway in the maintenance of inflammation and joint pain in CIA rats, further 16 rats were randomly assigned to two groups after inducing the CIA model: the CIA + vehicle group (CIA rats treated with normal saline) and the CIA + OxPAPC group (CIA rats treated with the TLR2/TLR4 inhibitor OxPAPC) (*n* = 8 each group).

#### 2.3.3. Experiment 3

To determine the expression of TLR2 and TLR4 in synovial macrophages and synovial fibroblasts in the synovium tissue, we evenly separated 12 SD rats into the same three groups as in Experiment 1. The rats in the CIA + EA group received EA intervention as in Experiment 1 for two weeks.

### 2.4. Electroacupuncture (EA) Intervention and Sampling in Experiment 1

**Figure 1:**
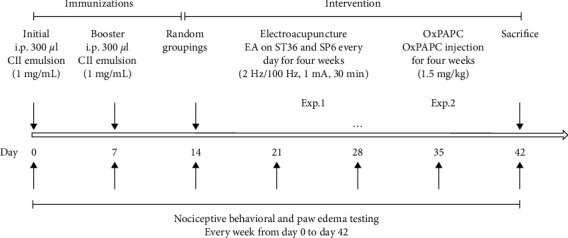
Experimental design for the effect of EA on CIA rats (experiment 1 and experiment 2). Male SD rats were initially immunized at the base of the tail on day 0 and boosted on day 7. From day 14 to day 42, rats in the CIA + EA group and the CIA + OxPAPC group received EA intervention or inhibitor injection every day, respectively. From day 0 to day 42, paw edema and pain responses were tested weekly in each group of rats.

Rats in the CIA + EA group received EA intervention at ST36 (located about 5 mm inferior to the capitulum fibulae and posterolateral to the hindlimb knee joint) and SP6 (located at the medial side of the hind leg, 10 mm directly above the tip of the medial malleolus) on day 14 after the initial immunization. Briefly, rats were lightly anesthetized with isoflurane (1.5% in oxygen) delivered via an anesthesia unit (Matrix Company, Midmark Animal Health, Versailles, OH, USA) and underwent EA stimulation by inserting needles (0.5 × 32 mm, Suzhou, China) at ST36 and SP6 in a depth of about 5 mm and 3 mm, respectively. The inserted needles were further connected to an electronic acupuncture treatment instrument (Hans-100A, Nanjing Jisheng Medical Technology, Co., Ltd., China). The EA parameters were alternating frequency of 2 Hz/100 Hz, a pulse width of 0.2–0.6 ms, and an intensity of 1 mA. The stimulation procedure was performed for 30 min every day for 28 consecutive days (Figure 1). Rats in the control group and the CIA group were anesthetized in the same manner as those in the CIA + EA group but did not receive EA stimulation.

Upon completion of EA intervention, all rats were anesthetized with pentobarbital sodium (50 mg/kg, i.p.) for blood collection and then sacrificed through decapitation (on day 42 after the first immunization). Blood (*n* = 12 per group) was taken via the tail vein and collected into procoagulation tubes (BD Vacutainer, Franklin Lakes, New Jersey, USA) for the separation of serum. Then, it was divided into aliquots and kept under −80°C for serological determinations. The ankle joints of hind paws (*n* = 6 per group) were separated for further histopathological analysis. The ankle synovial membrane tissues of hind paws (*n* = 6 per group) were isolated and kept under −80°C for further western blotting analysis.

### 2.5. Paw Edema Analysis

The paw edema of each rat's bilateral hind limbs (up to the ankle joint) was examined once a week from day 0 to day 42 (Figure 1) with a water displacement plethysmometer (Ugo Basile, Comerio, Varese, Italy). The mean values of water displacement volume were calculated from 3 measurements to judge the extent of paw edema [29].

### 2.6. Nociceptive Behavioral Tests

The pain responses of each rat's bilateral hind paws, including mechanical withdrawal threshold (MWT) and thermal withdrawal latency (TWL), were measured once a week from day 0 to day 42 (Figure 1). MWT was examined by electronic Von Frey (38450, Ugo Basile, Comerio, Varese, Italy). TWL was examined by a thermal plantar analgesia instrument (37370, Ugo Basile, Comerio, Varese, Italy) [30], with a heat intensity of 50 units and a cut-off time of 30 s. Mean MWT and TWL were calculated from 3 individual tests with a 5-min interval to represent the mechanical allodynia and thermal hyperalgesia.

### 2.7. Radiographic and Histopathological Analysis of Ankle Joints

For radiographic analysis, the hind limbs were imaged on a multimodality in vivo imaging system (Kodak Company, Rochester, NY, USA) shortly before the rats were sacrificed. The exposure time was 10 s, and the f-stop factor was 2.5.

For histopathological analysis, the protocol was performed as previously described [31]. Briefly, the fresh ankle joints were fixed with 4% paraformaldehyde (Servicebio, Wuhan, China) and then decalcified with 10% ethylene diamine tetraacetic acid (EDTA, Servicebio, Wuhan, China) at room temperature for 40 days. The processed joints were dehydrated, embedded, and sectioned at 4 *μ*m thickness in a sagittal plane. Sections were then stained with hematoxylin-eosin (HE) for assessing EA's effect on the histopathological damage in joints. Images were acquired and observed under an upright light microscope (Nikon Inc., Tokyo, Japan). HE staining score was evaluated by an investigator who was blinded to the experimental protocol. The following morphological criteria were considered as follows [32]: score 0, no damage; score 1, edema; score 2, presence of inflammatory cells; score 3, bone resorption.

### 2.8. Serum Cytokine Levels Detection

Serum TNF-*α*, IL-1*β*, and IL-6 were measured by Meso Scale Discovery (MSD) electrochemiluminescence technology with a V-PLEX custom rat cytokine kit (K153AOH-1, MSD, Rockville, Maryland, USA) according to the manufacturer's instructions [33]. In short, the 96-well plate was washed 3 times with 150 *μ*l wash buffer. After the washing, wells were incubated with 50 *μ*l serum sample for 2 h at room temperature with shaking. Twenty-five*μ*l of detection antibody solution was then added and incubated for 2 h at room temperature with shaking. After adding 150 *μ*l of 2 × read buffer *T* to each well, the 96-well plate was detected with MESO QuickPlex SQ 120 machine (MSD, Rockville, Maryland, USA), and data were analyzed via MSD Discovery Workbench software version 4.0.12.

### 2.9. Western Blotting Analysis

For western blotting analysis, total proteins were obtained from the ankle synovium tissues with RIPA lysis buffer (Beyotime Biotechnology, Shanghai, China), containing 1% PMSF. Protein concentrations were quantitatively determined with a BCA protein assay kit (Beyotime Biotechnology, Shanghai, China). Proteins were separated by 10% SDS-PAGE gel, transferred onto a 0.45 *μ*m PVDF membrane (Millipore, Burlington, Massachusetts, USA), and blocked with 5% BSA (Amresco, Solon, Ohio, USA). After the blocking, the membranes were blotted with primary antibodies overnight at 4°C as follows: anti-TLR2 (sc-21760, Santa Cruz, Dallas, Texas, USA), anti-TLR4 (sc-293072, Santa Cruz, Dallas, Texas, USA), anti-MyD88 (4283, Cell Signaling Technology, Danvers, Massachusetts, USA), anti-Phospho-NF-*κ*B p65 (3033S, Cell Signaling Technology, Danvers, Massachusetts, USA), and anti-*β*-actin (4970, Cell Signaling Technology, Danvers, Massachusetts, USA) and then incubated with the corresponding secondary antibodies (Jackson, ImmunoResearch Laboratories, West Grove, PA, USA) for 2 h at room temperature. Protein visualization was fulfilled with the enhanced chemiluminescence reagents (Millipore, Burlington, Massachusetts, USA) on a gel imaging system (Tanon Science and Technology Co., Ltd., Shanghai, China). Band density (quantification) was determined through TotalLab Quant analysis software (TotalLab Limited, England) after the subtraction of the background and normalization against *β*-actin.

### 2.10. Drug Administration in Experiment 2

Oxidized 1-palmitoyl-2-arachidonyl-sn-glycero-3-phosphorylcholine (OxPAPC) (tlrl-oxp1, InvivoGen, San Diego, USA), a dual TLR2 and TLR4 inhibitor, was dissolved in normal saline (1 mg/ml). Rats in the CIA + OxPAPC group and the CIA + vehicle group in Experiment 2 were administrated with OxPAPC (1.5 mg/kg) and normal saline, respectively, via the tail vein injection once a day for 4 consecutive weeks.

### 2.11. Double-Immunofluorescence Labeling in Experiment 3

Serial 4-*μ*m-thick paraffin sections from CIA synovial tissues were deparaffinized in xylene and rehydrated through a graded ethanol series. Then, the sections were immersed in the 0.01 M citrate buffer (pH 6.0), and microwave irradiation was performed three times (8 min/time) for antigen retrieval. The sections were incubated in 5% goat serum for 1 h before immunostaining. In double-labeling experiments, the sections were incubated with anti-CD68 (pAb, 1:200, ab125212, Abcam, British; mAb, 1:100, NBP2-32831, NOVUS, USA), anti-Vimentin mAb (1:200, ab92547, Abcam, British; 1 : 200, ab20346, Abcam, British), anti-TLR2 pAb (1:150, 17236-1-AP, Proteintech, China), and anti-TLR4 mAb (1 : 100, sc-293072, Santa Cruz, USA) at 4°C overnight, followed by incubated with secondary antibodies, goat anti-rabbit IgG H&L(FITC) (1 : 200, ab6717, Abcam, British) or goat anti-mouse IgG H&L (Cy3) (1:200, ab97035, Abcam, British). The poststaining sections were examined with a full-spectrum scanning confocal microscope (FV1200, Olympus, Japan), and pairs of images were superimposed for colocalization analysis.

### 2.12. Statistical Analysis

Data were reported as mean ± standard deviation (SD) and were examined by one-way ANOVA, followed by the least significant difference (LSD) test for comparisons among multiple groups. The LSD test was run only if *F*-value achieved *P* < 0.05, and there was no significant variance in homogeneity. *P* < 0.05 was considered statistically significant. All analyses were performed using Statistical Package for the Social Sciences (SPSS) version 23.0 (SPSS Inc., Chicago, Illinois, USA).

## 3. Results

### 3.1. EA Intervention Reduced CIA-Induced Hind Paw Edema

**Figure 2:**
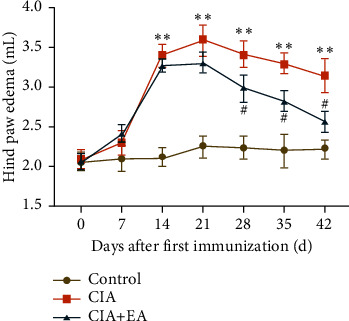
Measurement of the hind paw edema once a week from the day of the first immunization to the end of the EA intervention. (Data were expressed as mean ± SD, *n* = 12 per group, ^*∗∗*^*p* < 0.01 compared with the control group, ^#^*p* < 0.05 compared with the CIA group).

The hind paw edema was employed for the assessment of EA's effect on RA progression. As expected, the hind paw edema in the CIA group obviously increased from day 14 after the first immunization and lasted to day 42 compared with that in the control group (Figure 2, *p* < 0.01). EA stimulation at ST36 and SP6 showed marked alleviation of paw edema in the CIA + EA group after two weeks of EA intervention compared with that in the CIA group (Figure 2, *p* < 0.05).

### 3.2. EA Intervention Attenuated Allodynia and Hyperalgesia in CIA

**Figure 3:**
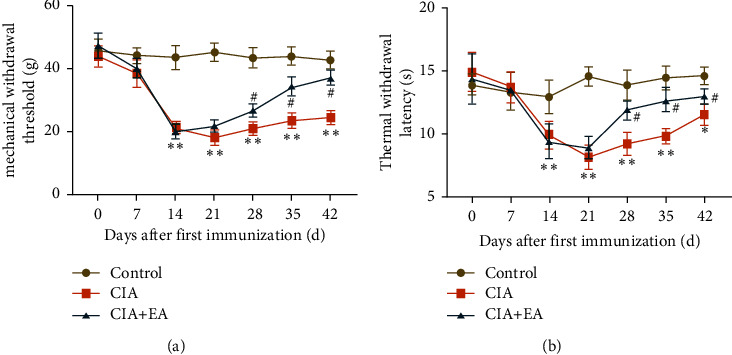
Measurement of the hind paw: (a) mechanical withdrawal threshold and (b) thermal withdrawal latency once a week from the day of the first immunization to the end of the EA intervention. (Data were expressed as mean ± SD, *n* = 12 per group, ^*∗*^*p* < 0.05, ^*∗∗*^*p* < 0.01 compared with the control group, ^#^*p* < 0.05 compared with the CIA group).

To explore the analgesic effects of EA, MWT and TWL were detected in all groups of rats. After successful induction of CIA (day 14 after the first immunization), MWT in the CIA group was much less than that in the control group with marked differences (Figure 3(a), *p* < 0.01). However, in comparison with the CIA group, MWT started to prominently recover in the CIA + EA group after two weeks of EA stimulation at ST36 and SP6 (Figure 3(a), *p* < 0.05). Similar results were corroborated in TWL detection (Figure 3(b), *p* < 0.01, *p* < 0.05). These results implicated that the long-term EA intervention may attenuate the arthritic inflammatory hyperalgesia in CIA rats.

### 3.3. EA Intervention Improved Histopathological Lesions in the Ankle Joint of CIA Rats

**Figure 4:**
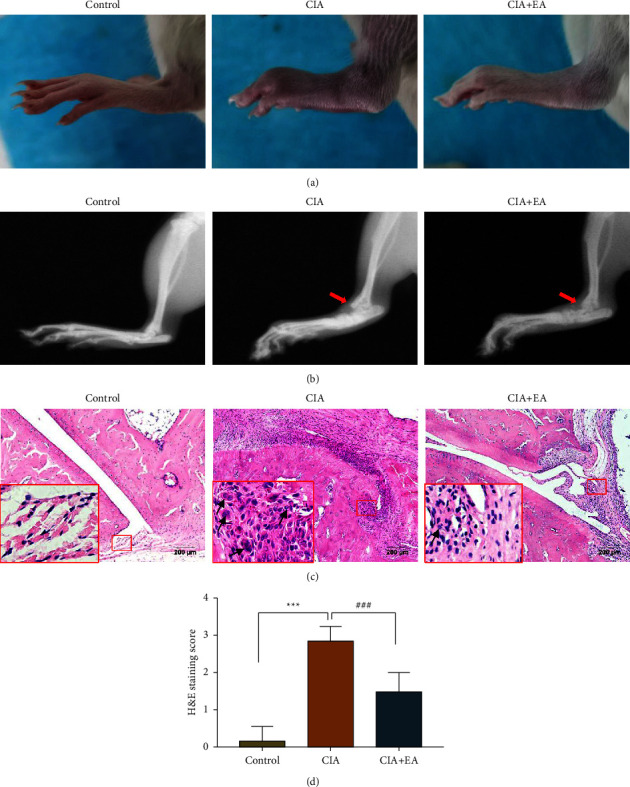
Macroscopic morphology, radiographic, and histopathological analysis of ankle joints. (a) Typical images of hind paws from each group after 28 days' EA intervention. *n* = 12 per group. (b) Representative radiographic images of hind paws from each group after 28 days' EA intervention. Red arrows indicate the bone erosion regions in the ankle joints, *n* = 12 per group. (c) HE staining of ankle joints from each group after 28 days' EA intervention. Black arrows indicate the infiltrated inflammatory cells, *n* = 6 per group. Scale bar: 200 *μ*m. (d) HE staining score of each group. (Data were expressed as mean ± SD, *n* = 6 per group, ^*∗∗∗*^*p* < 0.001 compared with the control group, ^###^*p* < 0.001 compared with the CIA group).

On day 42 after the first immunization, the visual redness and swelling of the hind paws in the CIA + EA group showed a great relief compared with those in the CIA group (Figure 4(a)).

As shown in the representative radiographs, bone erosion in the ankle joint was exacerbated in the CIA group compared to the control group whereas EA could distinctly reduce articular bone destruction and reverse the above trend in CIA rats (Figure 4(b)).

Consistent with the clinical behaviors, histopathological observations of ankle joints revealed signs of severest arthritis in the CIA rats. There was no evidence of inflammatory activity or joint erosion in the control group, while the massive inflammatory cell infiltration, synovial hyperplasia, and joint erosion were presented in the CIA group. The histological damage scores were higher in the CIA group (Figure 4(d), *p* < 0.001). After 28 days of EA intervention, these histopathological features significantly ameliorated in the CIA + EA group, and the HE staining score of the CIA + EA group also decreased remarkably (Figures 4(c) and 4(d), *p* < 0.001).

### 3.4. EA Intervention Showed the Anti-Inflammatory Effect on CIA Rats

**Figure 5:**
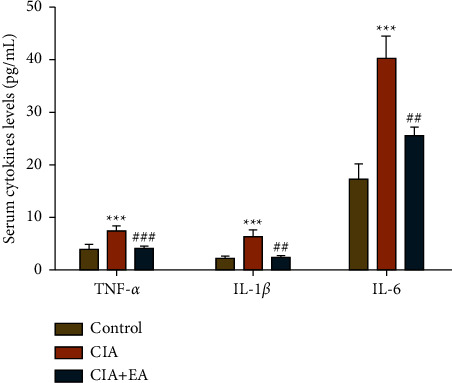
EA intervention inhibited expression of serum pro-inflammatory cytokines. The contents of TNF-*α*, IL-1*β*, and IL-6 were detected with MSD technology. (Data were expressed as mean ± SD, *n* = 12 per group, ^*∗∗∗*^*p* < 0.001 compared with the control group, ^##^*p* < 0.01, ^###^*p* < 0.001 compared with the CIA group).

It is well documented that pro-inflammatory cytokines play a vital role in the maintenance of chronic inflammation and tissue damage during RA progression. MSD test was conducted to assess the contents of serum TNF-*α*, IL-1*β*, and IL-6. As shown in Figure 5, compared with those in the control group, contents of serum TNF-*α*, IL-1*β*, and IL-6 noticeably elevated in the CIA group (5, *p* < 0.001). On the contrary, the serum levels of these cytokines significantly decreased in the CIA + EA group after 28 days' EA intervention (Figure 5, *p* < 0.01, *p* < 0.001).

### 3.5. EA Intervention Inhibited the Activation of TLR2/4-MyD88-NF-Κb Signaling Pathway in CIA

**Figure 6:**
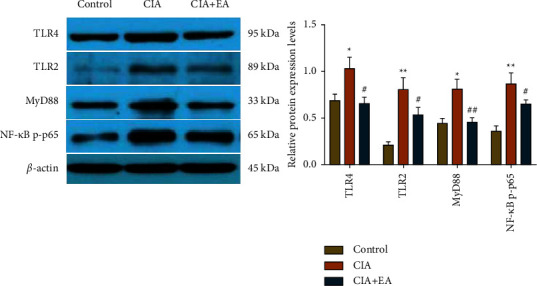
EA intervention inhibited TLR2/4-MyD88-NF-*κ*B pathway. The expression levels of TLR2, TLR4, MyD88, and NF-*κ*B p-p65 derived from ankle synovium were analyzed through western blotting. (Data were expressed as mean ± SD, *n* = 6 per group, ^*∗*^*p* < 0.05, ^*∗∗*^*p* < 0.01 compared with the control group, ^#^*p* < 0.05, ^##^*p* < 0.01 compared with the CIA group).

To further elucidate the underlying molecular mechanisms of analgesic and anti-inflammatory effects of EA intervention, we adopted western blotting to detect the expression levels of key proteins in the TLR2/4-NF-*κ*B pathway in the ankle synovium. The results indicated that expression levels of TLR2, TLR4, MyD88, and NF-*κ*B p-p65 in the CIA group were significantly upregulated by different degrees in comparison with those in the control group (Figure 6, *p* < 0.05, *p* < 0.01) whereas those in the CIA + EA group were remarkably downregulated compared with those in the CIA group (Figure 6, *p* < 0.05, *p* < 0.01). This illustrated that EA at ST36 and SP6 may ameliorate inflammation by inhibiting the TLR2/4-MyD88-NF-*κ*B pathway.

### 3.6. Blockade of TLR2/4 Signaling Mimicked the Analgesic and Anti-Inflammatory Effects of EA in CIA Rats

**Figure 7:**
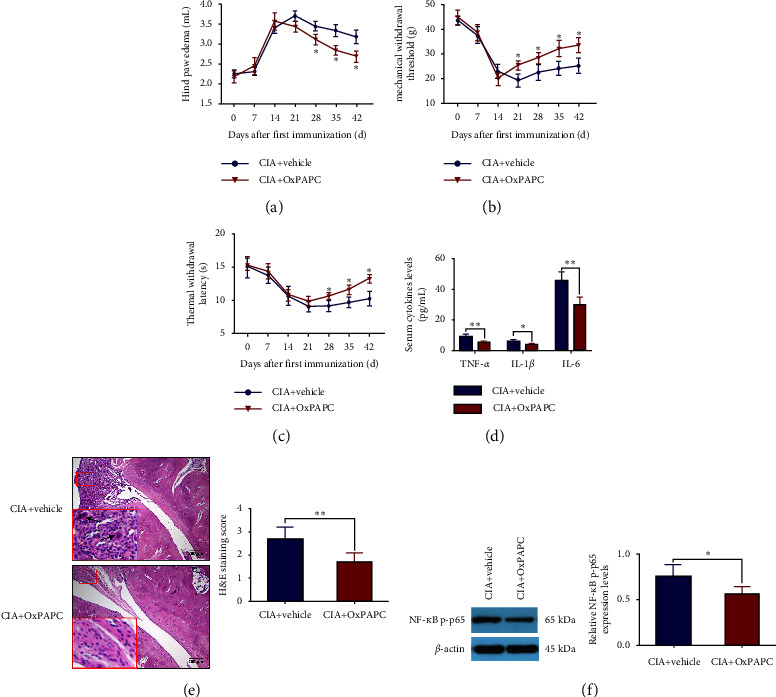
OxPAPC intervention relieved the inflammation and joint pain in CIA rats. (a) Measurement of the hind paw edema once a week from day 0 to day 42. Measurement of the hind paw (b) mechanical withdrawal threshold and (c) thermal withdrawal latency once a week from day 0 to day 42. (d) The contents of TNF-*α*, IL-1*β*, and IL-6 in serum. (e) Histopathological analysis of ankle joints. Scale bar: 200 *μ*m. (f) The expression levels of NF-*κ*B p-p65 in ankle synovium. (Data were expressed as mean ± SD, *n* = 8 per group in Figure (a–d), *n* = 4 per group in Figure (e and f), ^*∗*^*p* < 0.05, ^*∗∗*^*p* < 0.01 compared with the CIA + vehicle group).

To further assess whether the anti-inflammatory and analgesic effects of EA were mediated by the TLR2/4 signaling pathway, a dual TLR2 and TLR4 inhibitor, OxPAPC, was administered to CIA rats. Subsequently, behavioral tests and experiments such as and WB were conducted. The results indicated that the hind paw edema in the CIA + OxPAPC group decreased from day 28 to day 42 compared with that in the CIA + vehicle group (Figure 7(a), *p* < 0.05). Rats in the CIA + OxPAPC group had significantly increased MWT and TWL compared to rats in the CIA + vehicle group but significantly lower serum levels of TNF-*α*, IL-1*β*, and IL-6 (Figures 7(b)–7(d), *p* < 0.05, *p* < 0.01). Histopathological observations showed visible inflammatory cell infiltration and synovial hyperplasia in the CIA + vehicle group, while the pathological changes in the ankle joint of rats in the CIA + OxPAPC group were significantly improved (Figure 7(e), *p* < 0.01). Sure enough, the expression levels of NF-*κ*B p-p65 were notably downregulated in the CIA + OxPAPC group (Figure 7(f), *p*  < 0.05). All these results suggested that OxPAPC could relieve the inflammation and joint pain in CIA rats, inversely verifying that the TLR2/4 signaling pathway plays an important role in the EA treatment of CIA.

### 3.7. EA Intervention Suppressed the Expression of TLR2 and TLR4 on Synovial Fibroblasts and Macrophages in Synovium

**Figure 8:**
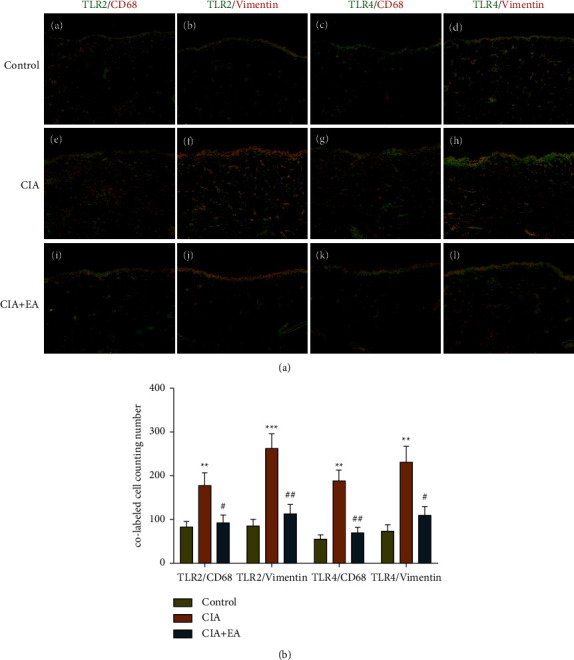
EA intervention decreased the expression of TLR2 and TLR4 on synovial fibroblasts and macrophages. (a) Synovium sections were double immunofluorescence stained for TLR2 or TLR4 and CD68 (macrophages marker) or Vimentin (fibroblasts marker), respectively; TLR2 and TLR4 immunopositive cells appear in green color, CD68 and Vimentin immunopositive cells appear in red color, and colabled cells appear in yellow color. The magnification of immunofluorescent images is 200x. (b) The number of double staining cells of TLR2 or TLR4 and CD68 or Vimentin. (Data were expressed as mean ± SD, *n* = 4 per group, ^*∗∗∗*^*p* < 0.001, ^*∗∗*^*p* < 0.01 compared with the control group; ^##^*p* < 0.01, ^#^*p* < 0.05 compared with the CIA group).

To characterize the TLR2 and TLR4 expressing cells in the joint synovium, tissue sections were double immunofluorescence stained for TLR2 or TLR4 and the macrophages marker CD68 or the fibroblasts marker Vimentin, respectively.  Figure 8 shows representative staining patterns of CD68 or Vimentin and TLR2 or TLR4 expression in the synovium tissue. In all three groups, the majority of TLR2 and TLR4 expression was detected on macrophages and synovial fibroblasts in the lining layer and sublining layer of the synovium, and in particular, there were pronounced TLR2 and TLR4 expression in the synovial lining layer. Compared with the control group, the number of positive staining cells for TLR2 and TLR4 on synovial fibroblasts and macrophages in synovial lining and sublining layers was significantly increased in CIA rats (Figure 8). EA intervention significantly reduced the number of positive staining cells for TLR2 and TLR4 on synovial fibroblasts and macrophages in synovium tissues (Figure 8).

## 4. Discussion

In the present study, an experimental RA model of the rat was established through immunization with CII collagen, a major component of hyaline cartilage, combined with CFA, which is the most commonly used method for developing an autoimmune model of RA [26]. Our results showed that CIA rats exhibited severe evoked joint pain and paw edema compared with rats in the control group. Radiographic and histopathological analysis confirmed the obvious pathological changes such as degeneration of joint structures, synovial hyperplasia, inflammatory cell infiltration, and bone erosion in the arthritic joints of CIA rats. Also, the contents of pro-inflammatory cytokines TNF-*α*, IL-1*β*, and IL-6 in serum were significantly elevated, and the expression levels of TLR2, TLR4, MyD88, and NF-*κ*B p-p65 were significantly upregulated in the synovium of CIA rats. These are consistent with the previous results obtained from patients with RA [34, 35] and experimental RA model animals [36–39], indicating that CIA rats showed significant inflammatory responses and hyperalgesia at arthritic joints, accompanied by the enhanced activation of the TLR2/4 signaling in the synovium.

Acupuncture is one of the oldest therapeutic strategies in the world and now has been widely reported to possess analgesic and anti-inflammatory effects in autoimmune diseases [40, 41]. In clinical practice, ST36 is the most frequently used acupuncture point for treating RA [42]. And in the studies of acupuncture effects on experimental RA animals, ST36 is also commonly used [43–45]. Meanwhile, a systemic review has shown that ST36 alone or combined with other acupoints is beneficial to the clinical status of RA [7]. Therefore, in this study, we selected two acupoints, ST36 and SP6, for EA intervention. The results showed that EA at ST36 and SP6 had a distinct anti-inflammatory and analgesic effect on CIA rats, as evidenced by reduced articular swelling, improved arthro-pathology, decreased levels of pro-inflammatory cytokines, and relieved mechanical allodynia and thermal hyperalgesia.

The synovium is the principal target tissue of inflammation in RA [46], and persistent chronic inflammation of the synovial membrane in RA causes inflammatory pain, which is known to be difficult to treat [47]. Emerging studies have indicated that EA has significant analgesic effects on inflammatory pain in CIA rats, which is related to the central descending pain inhibitory system mediated by adrenergic, cholinergic, and serotonergic receptors [48, 49]. However, it has been reported that pro-inflammatory cytokines, such as TNF-*α*, IL-1*β*, and IL-6, could directly increase the sensitivity and excitability of the primary afferents in the inflamed joints, leading to both peripheral and central sensitization [50, 51]. Our results also showed a positive correlation between nociceptive behavioral responses and articular inflammation in CIA rats. Therefore, inhibiting the secretion of pro-inflammatory cytokines in RA can effectively relieve joint pain.

Over the past two decades, increasing data have supported the critical role of the innate immune system in the pathogenesis of RA [52, 53]. Specifically, TLRs, the key recognition structures of the innate immune system, can recognize microbial products and endogenous ligands released upon cell damage and necrosis and are expressed on cells within the RA joint [20, 54]. Meanwhile, a variety of endogenous TLR ligands, including heat shock proteins, high mobility group box-1 protein (HMGB1), host DNA, fibrinogen, and tenascin-C, have been identified in the synovium of patients with RA, predominantly TLR2 and/or TLR4 agonists [55, 56]. The functions of TLR2/4 signaling have been extensively studied in RA. A large body of evidence obtained from *in vivo* animal models and *in vitro* human explants has confirmed that the activation of TLR2 and TLR4 by endogenous TLR ligands in arthritic joints can trigger the innate immune response and initiate the production of pro-inflammatory cytokines, chemokines, and proteases, which are proposed to be responsible for the perpetuating inflammation and joints destruction of RA [22, 57–60]. Therefore, the pivotal role of TLR2/4 signaling in the pathogenesis of RA had been well established, and our study focused on the modification of the TLR2/4 signaling pathway in EA intervention.

At present, the development of promising therapeutic strategies for the treatment of RA targeting TLRs is emerging [61]. Several preclinical studies have shown that the blockade of TLR2 significantly inhibited the production of pro-inflammatory cytokines TNF-*α*, IL-1*β*, and IL-6 in cultured synoviocytes from RA patients [62]. Inhibition of TLR4 not only alleviated the severity of experimental arthritis and suppressed IL-1*β* expression in arthritic joints of CIA mice [63] but also ameliorated inflammatory symptoms in adjuvant-induced arthritis (AIA) rat model and inhibited the secretion of IL-6 and IL-8 in both serum of the AIA rats and human synovial fibroblasts [64]. In addition, a recent study indicated that blocking TLR4 could effectively attenuate monoiodoacetate-induced arthritis in rats by relieving joint pain and reducing the expression of TNF-*α*, IL-1*β*, and matrix metallopeptidase-13 (MMP13) [65]. Similar to the results of these studies, our findings suggested that the blockade of TLR2/4 signaling by the TLR2/4 inhibitor OxPAPC could effectively alleviate articular inflammation and joint pain. Meanwhile, evidence from animal models suggested that EA can also alleviate arthritis by inhibiting TLR2 and/or TLR4 signaling [66, 67]. In line with these findings, our results indicated EA exerted anti-inflammatory and analgesic effects by inhibiting the inflammatory responses driven by the TLR2/4-MyD88-NF-*κ*B signaling pathway in the synovium of CIA rats.

The synovium is a connective tissue structure mainly comprised of resident macrophages and synovial fibroblasts [68]. The resident and infiltrating macrophages and the synovial fibroblasts are the principal innate immune effector cells of RA and are mainly activated by TLRs signaling [69, 70]. Biological agents targeting the pro-inflammatory cytokines TNF-*α* and IL-1*β*, predominantly produced by macrophages, have been proven clinically effective in RA [71]. Meanwhile, classical (M1, pro-inflammatory phenotype) macrophage activation occurs in the inflammatory environment of the RA joint dominated by TLRs signaling [72]. Numerous studies have confirmed that TLR2 and/or TLR4 were highly expressed on the synovial fibroblasts and triggered the production of the pro-inflammatory cytokines such as IL-6, chemokines, and tissue destroying mediators leading to inflammatory response and joints destruction [23, 59, 73–76]. In view of the important arthritic functions of TLRs signaling, especially TLR2 and TLR4 signaling in macrophages and synovial fibroblasts, we examined the effect of EA intervention on the expression of TLR2 and TLR4 on these two types of synovial cells of CIA rats. Our results showed that EA intervention markedly reduced the expression of TLR2 and TLR4 on macrophages and synovial fibroblasts in the synovial lining and sublining layers of CIA rats. In fact, the cellular and molecular mechanisms underlying the pathogenesis of RA are not fully understood by far, which undoubtedly increases the difficulty of elucidating the precise mechanism by which EA alleviates this disease. Inhibition of the innate immune response driven by TLRs signaling in the synovium seems to be the promising mechanism by which EA intervention alleviates the severity of RA, but the precise cellular and molecular mechanisms need to be further studied.

## 5. Conclusion

Taken together, the present findings indicate that EA markedly alleviated the severity of CIA in the rats by reducing paw swelling, serum levels of pro-inflammatory cytokines, and relieving joint pain. EA also can suppress the expression of TLR2/4-MyD88-NF-*κ*B signaling pathway-related proteins in the synovium, especially the TLR2 and TLR4 expression on synovial fibroblasts and macrophages, and inhibition of TLR2/4 signaling could mimic the analgesic and anti-inflammatory effects of EA. These data suggested that the anti-inflammatory and analgesic effects of EA treatment on RA are closely related to the inhibition of innate immunity-mediated inflammatory response in the macrophages and synovial fibroblasts driven by TLR2/4-MyD88-NF-*κ*B signaling pathway in the synovium.

## Data Availability

All data generated or analyzed during this study are included in this article.

## Abbreviations

EA: Electroacupuncture
CIA: Collagen-induced arthritis
SD: Sprague–Dawley
TNF-*α*: Tumor necrosis factor-alpha
IL-1*β*: Interleukin 1 beta
IL-6: Interleukin 6
RA: Rheumatoid arthritis
NSAIDs: Nonsteroidal anti-inflammatory drugs
DMARDs: Disease-modifying antirheumatic drugs
TLRs: Toll-like receptors
MyD88: Myeloid differentiation primary response 88
CII: Bovine type II collagen
CFA: Complete Freund's adjuvant
MWT: Mechanical withdrawal threshold
TWL: Thermal withdrawal latency
HE: Hematoxylin-eosin.

## References

[1] Ahlstrand I., Bjork M., Thyberg I., Borsbo B., Falkmer T. (2012). Pain and daily activities in rheumatoid arthritis. *Disability and Rehabilitation*.

[2] Mok C. C., Kwok C. L., Ho L. Y., Chan P. T., Yip S. F. (2011). Life expectancy, standardized mortality ratios, and causes of death in six rheumatic diseases in Hong Kong, China. *Arthritis and Rheumatism*.

[3] Mutru O., Laakso M., Isomaki H., Koota K. (1985). Ten year mortality and causes of death in patients with rheumatoid arthritis. *British Medical Journal*.

[4] Romao V. C., Lima A., Bernardes M., Canhao H., Fonseca J. E. (2014). Three decades of low-dose methotrexate in rheumatoid arthritis: can we predict toxicity?. *Immunologic Research*.

[5] Scott D. L., Wolfe F., Huizinga T. W. (2010). Rheumatoid arthritis. *The Lancet*.

[6] Seca S., Miranda D., Cardoso D. (2019). Effectiveness of acupuncture on pain, physical function and health-related quality of life in patients with rheumatoid arthritis: a systematic review of quantitative evidence. *Chinese Journal of Integrative Medicine*.

[7] Chou P. C., Chu H. Y. (2018). Clinical efficacy of acupuncture on rheumatoid arthritis and associated mechanisms: a systemic review. *Evidence-based Complementary and Alternative Medicine: eCAM*.

[8] Wu M. Y., Huang M. C., Liao H. H. (2018). Acupuncture decreased the risk of coronary heart disease in patients with rheumatoid arthritis in Taiwan: a Nationwide propensity score-matched study. *BMC Complementary and Alternative Medicine*.

[9] Lee H., Lee J. Y., Kim Y. J. (2008). Acupuncture for symptom management of rheumatoid arthritis: a pilot study. *Clinical Rheumatology*.

[10] Wang R., Jiang C., Lei Z., Yin K. (2007). The role of different therapeutic courses in treating 47 cases of rheumatoid arthritis with acupuncture. *Journal of Traditional Chinese Medicine*.

[11] Seca S., Patricio M., Kirch S., Franconi G., Cabrita A. S., Greten H. J. (2019). Effectiveness of acupuncture on pain, functional disability, and quality of life in rheumatoid arthritis of the hand: results of a double-blind randomized clinical trial. *Journal of Alternative and Complementary Medicine*.

[12] Choy E. (2012). Understanding the dynamics: pathways involved in the pathogenesis of rheumatoid arthritis. *Rheumatology*.

[13] Miossec P. (2004). An update on the cytokine network in rheumatoid arthritis. *Current Opinion in Rheumatology*.

[14] Taylor P. C. (2003). Anti-cytokines and cytokines in the treatment of rheumatoid arthritis. *Current Pharmaceutical Design*.

[15] Ouyang B. S., Gao J., Che J. L. (2011). Effect of electro-acupuncture on tumor necrosis factor-alpha and vascular endothelial growth factor in peripheral blood and joint synovia of patients with rheumatoid arthritis. *Chinese Journal of Integrative Medicine*.

[16] Gusmão J. N. F. M., Fonseca K. M., Ferreira B. S. P. (2021). Electroacupuncture reduces inflammation but not bone loss on periodontitis in arthritic rats. *Inflammation*.

[17] Li Q. H., Xie W. X., Li X. P. (2015). Adenosine A2A receptors mediate anti-inflammatory effects of electroacupuncture on synovitis in mice with collagen-induced arthritis. *Evidence-based Complementary and Alternative Medicine: eCAM*.

[18] Takeda K., Akira S. (2004). Toll-like receptors in innate immunity. *International Immunology*.

[19] Liu Y., Yin H., Zhao M., Lu Q. (2014). TLR2 and TLR4 in autoimmune diseases: a comprehensive review. *Clinical Reviews in Allergy and Immunology*.

[20] Thwaites R., Chamberlain G., Sacre S. (2014). Emerging role of endosomal toll-like receptors in rheumatoid arthritis. *Frontiers in Immunology*.

[21] Elshabrawy H. A., Essani A. E., Szekanecz Z., Fox D. A., Shahrara S. (2017). TLRs, future potential therapeutic targets for RA. *Autoimmunity Reviews*.

[22] Huang Q., Ma Y., Adebayo A., Pope R. M. (2007). Increased macrophage activation mediated through toll-like receptors in rheumatoid arthritis. *Arthritis and Rheumatism*.

[23] Kim K. W., Cho M. L., Lee S. H. (2007). Human rheumatoid synovial fibroblasts promote osteoclastogenic activity by activating RANKL via TLR-2 and TLR-4 activation. *Immunology Letters*.

[24] Joosten L. A. B., Koenders M. I., Smeets R. L. (2003). Toll-like receptor 2 pathway drives streptococcal cell wall-induced joint inflammation: critical role of myeloid differentiation factor 88. *The Journal of Immunology*.

[25] Choe J. Y., Crain B., Wu S. R., Corr M. (2003). Interleukin 1 receptor dependence of serum transferred arthritis can be circumvented by toll-like receptor 4 signaling. *Journal of Experimental Medicine*.

[26] Trentham D. E., Townes A. S., Kang A. H. (1977). Autoimmunity to type II collagen an experimental model of arthritis. *Journal of Experimental Medicine*.

[27] Kong X., Liu C., Zhang C. (2013). The suppressive effects of Saposhnikovia divaricata (Fangfeng) chromone extract on rheumatoid arthritis via inhibition of nuclear factor-*κ*B and mitogen activated proteinkinases activation on collagen-induced arthritis model. *Journal of Ethnopharmacology*.

[28] Brand D. D., Latham K. A., Rosloniec E. F. (2007). Collagen-induced arthritis. *Nature Protocols*.

[29] Yue M., Tao Y., Fang Y. (2019). The gut microbiota modulator berberine ameliorates collagen-induced arthritis in rats by facilitating the generation of butyrate and adjusting the intestinal hypoxia and nitrate supply. *The FASEB Journal*.

[30] Hargreaves K., Dubner R., Brown F., Flores C., Joris J. (1988). A new and sensitive method for measuring thermal nociception in cutaneous hyperalgesia. *Pain*.

[31] Kim Y. H., Kang J. S. (2015). Effect of methotrexate on collagen-induced arthritis assessed by micro-computed tomography and histopathological examination in female rats. *Biomolecules and therapeutics*.

[32] Du Z. H., Zhang C. W., Xie W. X. (2019). Adenosine A2A receptor mediates inhibition of synovitis and osteoclastogenesis after electroacupuncture in rats with collagen-induced arthritis. *Evidence-based Complementary and Alternative Medicine: eCAM*.

[33] Qi X., Yun C., Sun L. (2019). Publisher Correction: gut microbiota-bile acid-interleukin-22 axis orchestrates polycystic ovary syndrome. *Nature Medicine*.

[34] Mateen S., Zafar A., Moin S., Khan A. Q., Zubair S. (2016). Understanding the role of cytokines in the pathogenesis of rheumatoid arthritis. *Clinica Chimica Acta*.

[35] McInnes I. B., Schett G. (2007). Cytokines in the pathogenesis of rheumatoid arthritis. *Nature Reviews Immunology*.

[36] Liu W., Zhang Y., Zhu W. (2018). Sinomenine inhibits the progression of rheumatoid arthritis by regulating the secretion of inflammatory cytokines and monocyte/macrophage subsets. *Frontiers in Immunology*.

[37] Kwon H. K., Patra M. C., Shin H. J. (2019). A cell-penetrating peptide blocks Toll-likereceptor-mediated downstream signaling and ameliorates autoimmune and inflammatory diseases in mice. *Experimental and Molecular Medicine*.

[38] Takagi M. (2011). Toll-like receptor. *Journal of Clinical and Experimental Hematopathology*.

[39] Vargas-Ruiz R., Montiel-Ruiz R. M., Herrera-Ruiz M. (2020). Effect of phenolic compounds from Oenothera rosea on the kaolin-carrageenan induced arthritis model in mice. *Journal of Ethnopharmacology*.

[40] Gras M., Vallard A., Brosse C. (2019). Use of complementary and alternative medicines among cancer patients: a single-center study. *Oncology*.

[41] Huang Z., Hu Z., Ouyang J., Huang C. (2019). Electroacupuncture regulates the DREAM/NF-kappaB signalling pathway and ameliorates cyclophosphamide-induced immunosuppression in mice. *Acupuncture in Medicine*.

[42] Qi L., Tang Y., You Y. (2016). Comparing the effectiveness of electroacupuncture with different grades of knee osteoarthritis: a prospective study. *Cellular Physiology and Biochemistry*.

[43] Yim Y. K., Lee H., Hong K. E. (2007). Electro-acupuncture at acupoint ST36 reduces inflammation and regulates immune activity in Collagen-Induced Arthritic Mice. *Evidence-based Complementary and Alternative Medicine*.

[44] Zhu J., Su C., Chen Y., Hao X., Jiang J. (2019). Electroacupuncture on ST36 and GB39 acupoints inhibits synovial angiogenesis via downregulating HIF-1*α*/VEGF expression in a rat model of adjuvant arthritis. *Evidence-based Complementary and Alternative Medicine: eCAM*.

[45] Chen Y., Li H., Luo X. (2019). Moxibustion of Zusanli (ST36) and shenshu (BL23) alleviates cartilage degradation through RANKL/OPG signaling in a rabbit model of rheumatoid arthritis. *Evidence-based Complementary and Alternative Medicine: eCAM*.

[46] Orr C., Vieira-Sousa E., Boyle D. L. (2017). Synovial tissue research: a state-of-the-art review. *Nature Reviews Rheumatology*.

[47] Firestein G. S. (2003). Evolving concepts of rheumatoid arthritis. *Nature*.

[48] Baek Y. H., Choi D. Y., Yang H. I., Park D. S. (2005). Analgesic effect of electroacupuncture on inflammatory pain in the rat model of collagen-induced arthritis: mediation by cholinergic and serotonergic receptors. *Brain Research*.

[49] Park D. S., Seo B. K., Baek Y. H. (2013). Analgesic effect of electroacupuncture on inflammatory pain in collagen-induced arthritis rats: mediation by alpha2- and beta-adrenoceptors. *Rheumatology International*.

[50] Bas D. B., Su J., Wigerblad G., Svensson C. I. (2016). Pain in rheumatoid arthritis: models and mechanisms. *Pain Management*.

[51] Zhang A., Lee Y. C. (2018). Mechanisms for joint pain in rheumatoid arthritis (RA): from cytokines to central sensitization. *Current Osteoporosis Reports*.

[52] Gierut A., Perlman H., Pope R. M. (2010). Innate immunity and rheumatoid arthritis. *Rheumatic Disease Clinics of North America*.

[53] Edilova M. I., Akram A., Abdul-Sater A. A. (2021). Innate immunity drives pathogenesis of rheumatoid arthritis. *Biomedical Journal*.

[54] Huang Q. Q., Pope R. M. (2009). The role of toll-like receptors in rheumatoid arthritis. *Current Rheumatology Reports*.

[55] Goh F. G., Midwood K. S. (2012). Intrinsic danger: activation of Toll-like receptors in rheumatoid arthritis. *Rheumatology*.

[56] Piccinini A. M., Midwood K. S. (2010). DAMPening inflammation by modulating TLR signalling. *Mediators of Inflammation*.

[57] Huang Q. Q., Sobkoviak R., Jockheck-Clark A. R. (2009). Heat shock protein 96 is elevated in rheumatoid arthritis and activates macrophages primarily via TLR2 signaling. *The Journal of Immunology*.

[58] Kowalski M. L., Wolska A., Grzegorczyk J. (2008). Increased responsiveness to toll-like receptor 4 stimulation in peripheral blood mononuclear cells from patients with recent onset rheumatoid arthritis. *Mediators of Inflammation*.

[59] Kyburz D., Rethage J., Seibl R. (2003). Bacterial peptidoglycans but not CpG oligodeoxynucleotides activate synovial fibroblasts by toll-like receptor signaling. *Arthritis and Rheumatism*.

[60] Abdollahi-Roodsaz S., van de Loo F. A., van den Berg W. B. (2011). Trapped in a vicious loop: toll-like receptors sustain the spontaneous cytokine production by rheumatoid synovium. *Arthritis Research and Therapy*.

[61] Arleevskaya M. I., Larionova R. V., Brooks W. H., Bettacchioli E., Renaudineau Y. (2020). Toll-like receptors, infections, and rheumatoid arthritis. *Clinical Reviews in Allergy and Immunology*.

[62] Nic An Ultaigh S., Saber T. P., McCormick J. (2011). Blockade of Toll-like receptor 2 prevents spontaneous cytokine release from rheumatoid arthritis ex vivo synovial explant cultures. *Arthritis Research and Therapy*.

[63] Abdollahi-Roodsaz S., Joosten L. A. B., Roelofs M. F. (2007). Inhibition of Toll-like receptor 4 breaks the inflammatory loop in autoimmune destructive arthritis. *Arthritis and Rheumatism*.

[64] Samarpita S., Kim J. Y., Rasool M. K., Kim K. S. (2020). Investigation of toll-like receptor (TLR) 4 inhibitor TAK-242 as a new potential anti-rheumatoid arthritis drug. *Arthritis Research and Therapy*.

[65] Park H., Hong J., Yin Y. (2020). TAP2 a peptide antagonist of Toll-like receptor 4, attenuates pain and cartilage degradation in a monoiodoacetate-induced arthritis rat model. *Scientific Reports*.

[66] Dong Z. Q., Zhu J., Lu D. Z., Chen Q., Xu Y. L. (2018). Effect of electroacupuncture in Zusanli and kunlun acupoints on TLR4 signaling pathway of adjuvant arthritis rats. *American Journal of Therapeutics*.

[67] Ruan A., Wang Q., Ma Y. (2021). Efficacy and mechanism of electroacupuncture treatment of rabbits with different degrees of knee osteoarthritis: a study based on synovial innate immune response. *Frontiers in Physiology*.

[68] Smith M. D., Barg E., Weedon H. (2003). Microarchitecture and protective mechanisms in synovial tissue from clinically and arthroscopically normal knee joints. *Annals of the Rheumatic Diseases*.

[69] Drexler S. K., Kong P. L., Wales J., Foxwell B. M. (2008). Cell signalling in macrophages, the principal innate immune effector cells of rheumatoid arthritis. *Arthritis Research and Therapy*.

[70] Noss E. H., Brenner M. B. (2008). The role and therapeutic implications of fibroblast-like synoviocytes in inflammation and cartilage erosion in rheumatoid arthritis. *Immunological Reviews*.

[71] Feldmann M., Brennan F. M., Foxwell B. M., Taylor P. C., Williams R. O., Maini R. N. (2005). Anti-TNF therapy: where have we got to in 2005?. *Journal of Autoimmunity*.

[72] Cutolo M., Campitiello R., Gotelli E., Soldano S. (2022). The role of M1/M2 macrophage polarization in rheumatoid arthritis synovitis. *Frontiers in Immunology*.

[73] Agarwal S., Misra R., Aggarwal A. (2010). Induction of metalloproteinases expression by TLR ligands in human fibroblast like synoviocytes from juvenile idiopathic arthritis patients. *Indian Journal of Medical Research*.

[74] Pierer M., Rethage J., Seibl R. (2004). Chemokine secretion of rheumatoid arthritis synovial fibroblasts stimulated by Toll-like receptor 2 ligands. *The Journal of Immunology*.

[75] Philippe L., Alsaleh G., Pichot A. (2013). MiR-20a regulates ASK1 expression and TLR4-dependent cytokine release in rheumatoid fibroblast-like synoviocytes. *Annals of the Rheumatic Diseases*.

[76] Li Z., Cai J., Cao X. (2016). MiR-19 suppresses fibroblast-like synoviocytes cytokine release by targeting toll like receptor 2 in rheumatoid arthritis. *American Journal of Translational Research*.

